# A New Classification System for the Actions of IRS Chemicals Traditionally Used For Malaria Control

**DOI:** 10.1371/journal.pone.0000716

**Published:** 2007-08-08

**Authors:** John P. Grieco, Nicole L. Achee, Theeraphap Chareonviriyaphap, Wannapa Suwonkerd, Kamal Chauhan, Michael R. Sardelis, Donald R. Roberts

**Affiliations:** 1 Department of Preventive Medicine and Biometrics, Uniformed Services University of the Health Sciences, Bethesda, Maryland, United States of America; 2 Department of Entomology, Kasetsart University, Bangkok, Thailand; 3 Office of Disease Prevention and Control, Ministry of Public Health, Chiang Mai, Thailand; 4 Chemicals Affecting Insect Behavior Laboratory, United States Department of Agriculture, Beltsville, Maryland, United States of America; St. George's, University of London, United Kingdom

## Abstract

Knowledge of how mosquitoes respond to insecticides is of paramount importance in understanding how an insecticide functions to prevent disease transmission. A suite of laboratory assays was used to quantitatively characterize mosquito responses to toxic, contact irritant, and non-contact spatial repellent actions of standard insecticides. Highly replicated tests of these compounds over a range of concentrations proved that all were toxic, some were contact irritants, and even fewer were non-contact repellents. Of many chemicals tested, three were selected for testing in experimental huts to confirm that chemical actions documented in laboratory tests are also expressed in the field. The laboratory tests showed the primary action of DDT is repellent, alphacypermethrin is irritant, and dieldrin is only toxic. These tests were followed with hut studies in Thailand against marked-released populations. DDT exhibited a highly protective level of repellency that kept mosquitoes outside of huts. Alphacypermethrin did not keep mosquitoes out, but its strong irritant action caused them to prematurely exit the treated house. Dieldrin was highly toxic but showed no irritant or repellent action. Based on the combination of laboratory and confirmatory field data, we propose a new paradigm for classifying chemicals used for vector control according to how the chemicals actually function to prevent disease transmission inside houses. The new classification scheme will characterize chemicals on the basis of spatial repellent, contact irritant and toxic actions.

## Introduction

Science and society label almost any chemical used against insects as an “insecticide.” By definition, an insecticide (insect-icide or insect-icidal) is a chemical that kills insects. This single term is not adequate for meaningful discourse about chemicals, chemical actions, insect responses to chemicals, and the different ways in which chemicals are used. However, this single response is the foundation for the old paradigm that classifies chemicals sprayed on house walls for malaria control based solely on their killing action. A new paradigm is needed to take into account the behavioral actions of these chemicals in disrupting man-vector contact and thereby breaking disease transmission. The fact that repellent and irritant actions were first documented more than 60 years ago [1] but given no importance, illustrates how lack of appropriate labels and a conceptual framework of multiple chemical actions can work against knowledge and understanding. Today, any discussions about insecticides for malaria control operate under a de facto assumption that the chemical is toxic and it's only important function is to kill mosquitoes. As will be shown by research presented here, this assumption is wrong.

Over 45 years ago Dethier [2] showed that chemicals elicit multiple actions and that insects respond to those actions through a variety of behaviors. He noted that if we were to take a closer look at modes of action, we could find a much more diverse set of terms for oriented movements of insects toward or away from a chemical source. As early as 1953, Muirhead-Thomson [3] concluded chemicals could disrupt contact between humans and malaria-transmitting mosquitoes and stop disease transmission without killing the mosquitoes. Subsequent authors speculated that space repellents applied to house walls could have advantages over topical repellents on skin. In contrast to topical repellents, repellents designed for application on walls could be formulated for longer persistence and might even have a lower cost of production. Regardless, the search for alternative compounds has focused almost entirely on toxicity. Evidence that this search has not emphasized DDT's true mode of action is revealed by the fact that even now there are no labeled compounds for IRS use that elicit a spatial repellent response. Insecticides recommended for indoor residual spraying (IRS) continue to be evaluated almost entirely on mosquito mortality [4] and laboratory evaluations continue to use toxicity as the primary measure of success [5]–[7].

The overall aim of this research was to quantify and accurately describe chemical actions and mosquito responses to those actions using *Aedes aegypti* mosquitoes as a model system. Although *Ae. aegypti* does not transmit malaria, it is responsible for transmitting dengue and yellow fever viruses in urban environments. This species was selected as our model system because of its medical importance and because eggs can be stored dry and used when needed for producing test populations. Additionally, new colonies are easily established by bringing wild caught material from the field.

**Figure 1:**
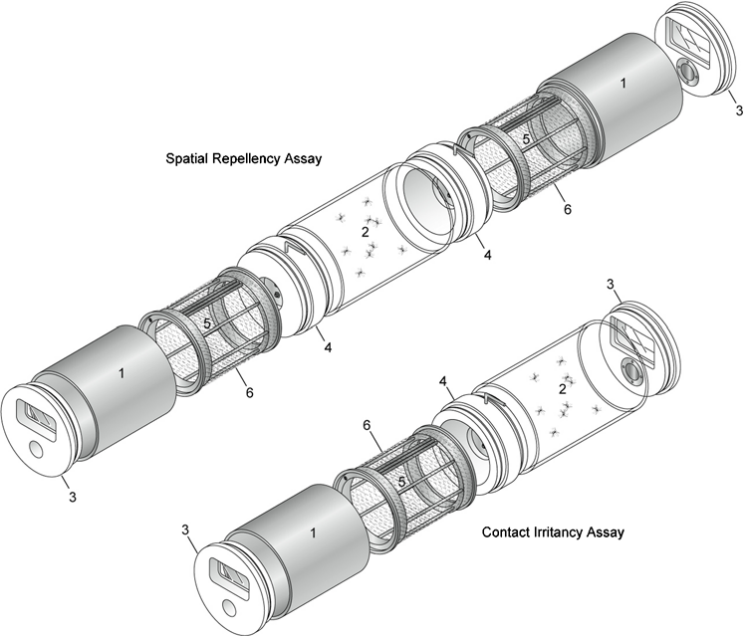
Schematic drawing of the high-throughput screening system showing the spatial repellency assay (top) and contact irritancy assay (bottom) assemblies. Major components include: 1, treatment (metal) cylinder; 2, clear (Plexiglas) cylinder; 3, end cap; 4, linking section; 5, treatment drum; and 6, treatment net.

We used a suite of assays to quantitatively characterize mosquito responses to toxic, contact irritant, and non-contact spatial repellent actions of insecticides [8] (Fig 1). These actions are defined in terms of the insect's response to the chemical. A toxic action produces knockdown or death after the mosquito makes physical contact with the chemical. A contact irritant action stimulates directed movement away from the chemical source after the mosquito makes physical contact. A spatial repellent action stimulates directed movement away from the chemical source without the mosquito making physical contact with the treated surface.

Thresholds exist for when and how insects respond to these chemical actions. These thresholds are governed by intrinsic and extrinsic factors such as inherent strength of a chemical action, chemical volatility, environmental temperature, humidity, proximity and length of exposure, and a mosquito's sensitivity to a compound, to name just a few factors. The dose dependent order in which thresholds are exceeded determines whether the primary mode of chemical action is repellent, irritant or toxicant. Research described here will show that house wall residues of three important and commonly used insecticides elicit varying combinations of behavioral and toxic actions. Based on these results, we are proposing criteria for revising classifications of chemicals that are presently recommended for use in malaria control programs. This revised classification scheme is a new paradigm for disease control that emphasizes a single or the combination of multiple chemical actions to control disease transmission by breaking man-vector contact. This new paradigm will permit chemists, toxicologists, and public health scientists to discuss and characterize chemicals according to their true modes of action.

## Methods

### Mosquitoes

Past research suggests that behavioral responses of extensively colonized mosquitoes to chemicals are diminished or non-existent. Therefore, we conducted all laboratory tests with female mosquitoes only recently brought from the field (i.e. F_1_ to F_3_). A colony of *Aedes aegypti* was maintained and renewed every 6 months with field populations from Thailand. The field populations were collected as larvae from Pateuy Village, Saiyok District, Kanchanaburi Province, western Thailand (14°20′11″N, 98°59′45″E). Baseline toxicity data revealed this population to be highly resistant to DDT, tolerant to alphacypermethrin and susceptible to dieldrin.

### Test compounds

Data presented here evolved from a larger ongoing research project to find a chemical that might be a cost-effective substitute for DDT in the control of malaria. Many insecticides were tested. Several of these are currently recommended for control of malaria. We found that the profiles of toxic, contact irritant, and spatial repellent actions varied widely with different chemicals. Of all the chemicals tested, we selected only three for testing in the field based on the distinct actions that were exhibited in the laboratory. Data will be presented on these chemicals alone. Based on laboratory tests, one of the three (dieldrin) was toxic but had no repellent or irritant actions. Another (alphacypermethrin) had irritant and toxic actions; but had no repellent action. The third chemical (DDT) exhibited all three actions; repellency, irritancy, and toxicity.

The test compounds were DDT (1,1 Bis(4-chlorophenyl)-2,2,2-trichloroethane, 98%)(Sigma-Aldrich), alphacypermethrin (a racemate comprising (S) alpha-cyano-3-phenoxybenzyl (1R, 3R)-3-(2,2 dichlorovinyl) 2,2-dimethyl cyclopropane carboxylate and (R) alpha-cyano-3-phenoxybenzyl (1S, 3S)-3-(2,2 dichlorovinyl) 2,2-dimethyl cyclopropane carboxylate)(BASF) and dieldrin (1,2,3,4,10,10-Hexachloro-1,4,4a,5,6,7,8,8a-octahydro-6,7-epoxy-1,4∶5,8-dimethanonaphthalene, 90%)(Sigma-Aldrich). Chemical solutions using an acetone solvent (1.5 ml) were applied evenly to nylon organdy netting strips (330 cm^2^) using a micropipette, resulting in treatment concentrations of 0.25, 2.5, 25, and 250 nmol/cm^2^. All netting, both control and treatment, were allowed to sit for at least 30 minutes to ensure that the acetone completely evaporated from the netting leaving only the chemical of interested (treatment) or the clean netting (control).

### Field netting treatment

We applied each compound in hut treatments at a dose that closely approximated the WHO recommended field application rate. In this case we applied alphacypermethrin at 2.5 nmoles/cm^2^ (0.03 g/m^2^ recommended use equates to 7.2 nmoles/cm^2^), DDT at 500 nmoles/cm2 (2.0 g/m^2^ recommended use equates to 564.2 nmoles/cm^2^) and dieldrin at 25 nmoles/cm2 (0.1 g/m^2^). Sheets of polyester netting(BioQuip®, Gardena, CA) (1 m×3 m) with mesh size of 24×20/inch were treated. Individual sheets of netting were saturated with treatment solution and excess solvent was allowed to evaporate.

### Laboratory Assays

A suite of assays (Fig 1) was developed which makes use of a single set of chambers configured in multiple ways to measure different actions: contact irritancy, spatial repellency and toxicity. Methodology employed to obtain each end point has been describe [8]. A brief description follows.

### Contact irritancy assay (CIA)

Assay is composed of a metal chamber that houses the netting which is connected to a clear receiving chamber. The two ends of the assay are separated by a beveled divider containing a butterfly valued gate. Ten mosquitoes were transferred into the treatment end of the assembly and, after 30 sec, the butterfly valve was opened. After 10 min, the valve was again closed, and counts were immediately made of the number of mosquitoes in the clear end (number escaping) and those remaining in the treatment end. Those knocked down were also recorded. For every two trials a control assay was run, in which the acetone-treated net was used in place of the insecticide treated one. Six replicates were performed at each treatment concentration.

### Spatial repellency assay

This assay consists of three chambers connected in unison. At one end is a treatment chamber and at the other end is a control chamber. Treatment and control chambers are connected to each other by a clear cylinder to form the complete spatial repellency assay assembly. Twenty mosquitoes were transferred into the clear (central) chamber, and the assay covered. After a 30 sec resting period, the butterfly valves were opened. After 10 min, the valves were closed and the number of mosquitoes in each chamber was counted as well as the numbers knocked down. Between replicates, the assay is disassembled to allow volatilized chemical to be ventilated from the chamber. Nine replicates were performed for each treatment concentration.

### Toxicity assay

A single chamber is used as an exposure chamber to evaluate a chemical's toxicity much in the same way as the bottle assay [7]. After preparing a chamber with treated netting, 20 mosquitoes were transferred into the chamber. After a 1 h exposure, the number of knocked down mosquitoes was recorded and all mosquitoes were transferred to holding cartons. These mosquitoes were provided a 10% sucrose-soaked cotton ball as a carbohydrate source and returned to the insectary. Mortality was recorded after 24 h. A control assay was included for all trials that have acetone-treated netting in the exposure chamber. Six replicates were performed at each treatment concentration.

### Field Studies

The field studies with experimental huts were conducted against F_1_ populations of *Ae. aegypti* in Thailand. These mosquitoes were 5–7 day old, mated females that were only provided a sugar meal prior to use in the field studies. These conditions were identical to those mosquitoes used in the laboratory assay. The goal was to confirm that the orderly sequence of chemical actions identified in laboratory tests would actually occur with natural mosquito population under field conditions. Two portable huts were constructed for evaluating entering and exiting behavior of *Ae. aegypti*. The huts were constructed in the fashion of indigenous Thai homes with wood walls and corrugated tin roof and were positioned 100 m from each other. The dimensions of the huts were 4 m wide×5 m long×3.5 m high with three windows and one door onto which could be affixed entrance and exit traps. Floors were adjusted and aligned with cement blocks and covered with a white sheet for detecting mosquitoes on the floor that had been knocked down. A series of aluminum panels were developed for holding treated netting which could be positioned around the interior surface of the hut. Each panel has a backing of aluminum wire mesh that prevents the netting from making contact with the hut wall.

### Hut Studies of Spatial Repellent Actions

Huts were fitted with window and door traps that were positioned inside to capture entering mosquitoes. Two pools of 100 mosquitoes were placed into two separate 1-gallon cardboard containers topped with mesh netting. One container was used for the treatment population and the other contained a control population. Populations were marked with luminous marking powder (BioQuip Products, Inc., Gardena CA.) using a ¼ in. paintbrush. The paintbrush was loaded with powder then quickly brushed against the mesh netting of the container lid in a circular motion from the outside circumference to the inside center of the container. Marked mosquitoes (100 per hut) were released 10 meters outside of each hut at 0540 hr just prior to sunrise and collections were made from the traps at 20 min intervals, from 0600–1800 h. Two collectors were positioned in each hut immediately after mosquitoes were released. All collected mosquitoes were placed in plastic cups and were labeled with time and location of each trap.

### Hut Studies of Contact Irritant Actions

Huts were armed with window and door traps placed outside to capture exiting mosquitoes. Marked mosquitoes (100 per hut) were released inside at 0540 hr. A human host entered an untreated bed net in each hut immediately after marked females were released indoors. All mosquitoes collected from the traps were placed in holding cups labeled by time and location. Removal of mosquitoes from the traps was made by collectors located outside the huts. At the top of each hour, the collectors located on the inside of the hut exited the bed net and searched the floor for knocked down mosquitoes. All cups were provided a 10% sugar soaked cotton pad and were checked after 24 hr to record mortality. Additional cups of 25 marked mosquitoes with moist sugar pads were placed in both huts as controls.

### Data analysis

Contact irritancy assay data were analyzed using the Wilcoxon two-sample test [9] for differences between numbers escaping from treated and control chambers. Spatial repellency assay data were analyzed by a nonparametric signed-rank test [9] to determine if the mean SAI (described below) for each treatment was significantly different from zero. For the toxicity data, percent knockdown and mortality values were corrected using Abbott's formula [10] and transformed to arcsine square root values for analysis of variance (ANOVA). For each chemical, knockdown and mortality at each treatment concentration was compared and separated using Tukey's honestly significant difference (HSD) test at *P* = 0.05 [11]. Means±SE of untransformed data were reported.

A spatial activity index (SAI), based upon the oviposition activity index of Kramer and Mulla [12], was used to evaluate the responses of female mosquitoes in the spatial repellency assay. We calculated the SAI for each experimental replication as SAI = (*N_c_*−*N_t_*)/(*N_c_*+*N_ t_*), in which *N_c_* was the number of females in the control chamber of the spatial repellency assay assembly and *N_t_* was the number of females in the treated chamber. The SAI was a measure of the proportion of females in the control chamber over the treated chamber after correcting for the proportion of females in the control chamber. The SAI varies from −1 to 1, with 0 indicating no response. An SAI value of −1 indicated that a greater proportion of mosquitoes moved into the treatment chamber than the control chamber thus indicating an attractant response. An SAI value of 1 indicated a greater proportion of mosquitoes moved into the control chamber (away from the treatment end of the assay device) indicating a repellent action.

Data from the field studies revealed that when releasing a fixed population, there are diminishing returns on the probability of recapture as higher numbers of marked mosquitoes are removed from the pool of potential responders. Therefore appropriate analysis of time-trend data of exiting mosquitoes from houses require adjustments for the numbers of mosquitoes capable of responding at a given time x. That is to say, the first half of the day is the richest period for showing the influence of chemical actions. The strength of the data declines as mosquitoes are removed from the population through recapture. Furthermore, in evaluating a contact irritant response the time when escape occurs is as important as the numbers that escape. The faster a mosquito escapes, the less is its chance of making lethal contact with a treated surface. The numbers that are knocked down in the hut must also be removed from total numbers in huts. Therefore, we focus our analysis on the first 7 hours and remove the numbers that are knocked down before they can escape.

## Results

### Contact Irritant Responses

**Figure 2:**
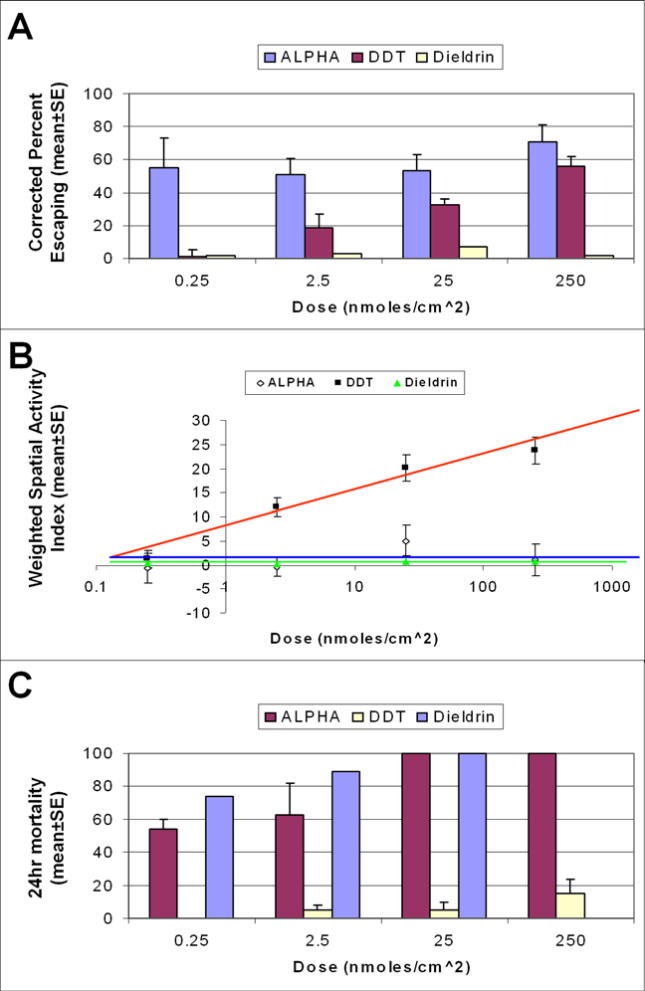
(A) Corrected percent escape (weighted based on percent responding), (B) Weighted spatial activity index, (C) Twenty four hour mortality for DDT, alphacypermethrin and dieldrin.

Our findings from laboratory tests for contact irritancy showed that the percent of *Ae. aegypti* females escaping from treatment chambers was proportional to the dose of insecticide used. In general, mean number and corrected percent escaping from treated chambers increased with increasing concentrations of the chemical treatment (Table 1). A significant (*P*<0.05) contact irritancy response to alphacypermethrin was observed at treatment concentrations of 0.25 nmoles/cm^2^ and higher. In other words, alphacypermethrin functioned as a contact irritant at all test concentrations. DDT produced significant contact irritancy responses at concentrations of 2.5 nmoles/cm^2^ and higher (Table 1). Dieldrin produced no contact irritant response at any of the doses tested. A side-by-side comparison of this dose response relationship for the three compounds can clearly be seen in Figure 2A.

**Table 1:** Response of female *Aedes aegypti* 1 in the contact irritancy assay to selected chemicals in the laboratory.

Chemical	Concentration (nmoles/cm2)	Number of trials (No. mosq.)	Number escaping (mean±SE)		Percent escaping_c_ 2 (mean±SE)	*P* 3
			Treated	Control		
DDT	0.25	6 (60)	0.0±0.0	0.5±0.3	−6±4	0.4545
	2.5	6 (60)	3.5±0.4	1.8±0.5	19±8	0.0519
	25	6 (60)	4.0±0.2	1.0±0.2	33±3	0.0022
	250	6 (60)	6.2±0.6	1.5±0.5	56±6	0.0022
α-cypermethrin	0.25	6 (60)	6.7±1.0	1.5±0.6	55±18	0.0087
	2.5	10 (100)	5.8±1.0	2.1±0.4	51±10	0.0119
	25	8 (80)	5.2±0.6	2.0±0.5	53±10	0.0016
	250	11 (110)	5.0±0.4	2.2±0.4	71±10	0.0001
dieldrin	0.25	6 (60)	0.2±0.2	0.7±0.2	−10±4	0.1515
	2.5	6 (60)	0.3±0.2	0.0±0.0	3±2	0.4545
	25	6 (60)	0.5±0.3	0.3±0.2	7±3	1.0000
	250	6 (60)	0.5±0.2	0.7±0.2	−2±4	1.0000

1 Four-7-d-old, non-bloodfed, THAI strain.

2 For each trial, percent escaping_c_ is percent escaping after correction using Abbott's formula.

3 *P* values are from Wilcoxon Two-Sample Test for difference between counts in the treated chamber versus count in the control chamber.

### Spatial Repellent Responses

The spatial repellent test, however, showed the mean percent responding was nearly uniform among treatment concentrations of alphacypermethrin, ranging from 8–20%, and the percent responding for dieldrin ranged from 7–17%. The mean percent responding to DDT showed an increase with increasing concentration, ranging from 7–29% at the two lowest concentrations and reaching 33–55% at the two higher concentrations (Table 2). No statistically significant spatial repellent response was documented for any treatment concentration of alphacypermethrin. Dieldrin, also, produced no statistically significant spatial repellent response at any dose tested. In contrast, DDT showed significance at 2.5 nmole/cm^2^ (*P* = 0.0010), 25 nmoles/cm^2^ (*P* = 0.0005) and 250 nmoles/cm^2^ (*P* = 0.0039). To more accurately express the intensity of the directional movement, a weighted spatial activity index was calculated by factoring in the percent responding. These weighted values showed an increased response to increasing doses of DDT as well as directional movement away from the treated cylinder. With alphacypermethrin, there was no statistical significance in directional movement or increase in response with increasing concentrations (Fig 2B).

**Table 2:** Response of female *Aedes aegypti* 1 in the spatial repellency assay to selected chemicals in the laboratory.

Chemical	Concentration (nmoles/cm^2^)	*n* 2	Mean percent responding (SE)	Mean SAI^3^ (SE)	SR^4^	*P*>*S*
DDT	0.25	9	7 (2)	−0.05 (0.21)	−1.0	1.0000
	2.5	12	29 (5)	0.62 (0.12)	38.0	0.0010
	25	12	33 (1)	0.62 (0.07)	39.0	0.0005
	250	9	53 (6)	0.49 (0.05)	22.5	0.0039
α-cypermethrin	0.25	9	12 (2)	−0.04 (0.23)	−0.5	1.0000
	2.5	9	8 (4)	−0.07 (0.12)	0.0	1.0000
	25	10	15 (3)	0.16 (0.23)	6.5	0.4844
	250	9	20 (2)	−0.13 (0.21)	−5.5	0.5625
dieldrin	0.25	9	12 (5)	0.25 (0.15)	5.5	0.1875
	2.5	9	7 (2)	−0.29 (0.22)	−7.0	0.4531
	25	9	17 (3)	−0.24 (0.22)	−7.0	0.2969
	250	9	11 (3)	0.02 (0.24)	0.5	1.0000

1 Four-7-d-old, non-bloodfed, THAI strain.

2 Twenty mosquitoes per trial.

3 SAI, spatial activity index.

4 SR, signed-rank statistic derived through PROC UNIVARIATE (SAS 1999).

### Toxic Responses

Of the three compounds, only alphacypermethrin gave consistent high levels (72–98% range) of knockdown at all treatment concentrations after a one hour exposure (Table 3). The two lowest concentrations of alphacypermethrin resulted in greater than 50% mortality after 24 hours (Fig 2C). One hundred percent mortality was obtained at the two higher doses. Dieldrin showed very low levels of knockdown after a one hour exposure but high levels of mortality (>70% at 0.25 nmoles/cm^2^ and 100% mortality at 25 nmoles/cm^2^). On the other hand, DDT showed very little if any knockdown (1–2%) at all test concentrations, and the knockdown that did occur was due to handling as exhibited by mortality in control populations. Low mortality was recorded for DDT at the highest concentration of 250 nmoles/cm^2^ (only 15% mortality after 24 hrs) (Fig 2C).

**Table 3:** Knockdown (KD) and adulticide activity(MORT) of DDT, alphacypermethrin and dieldrin against female *Aedes aegypti* 1 obtained from laboratory assays.

Chemical	Treatment (nmoles/cm2)	Number of trials (No. mosq.)	1 h KD^2^ (mean %±SE)	24 h MORT (mean %±SE)
DDT	0.25	3 (60)	2±2	0±0
	2.5	3 (60)	2±2	5±3
	25	3 (60)	0±0	5±5
	250	6 (120)	1±1	15±9
α-cypermethrin	0.25	6 (120)	73±13	54±6
	2.5	6 (120)	72±18	63±19
	25	6 (120)	98±1	100±0
	250	6 (120)	98±2	100±0
dieldrin	0.25	6 (120)	1±1	74±4
	2.5	6 (120)	2±1	89±5
	25	6 (120)	3±3	100±0

1 Four-7-d-old, non-bloodfed, THAI strain.

2 Knockdown and mortality of controls was <1% overall. na, not applicable.

### Confirmatory Field Studies

Baseline studies conducted prior to the addition of chemical to the interior of the huts demonstrated high between day variance and low same day variance (i.e. paired huts showed high variability from day to day while the huts showed identical patterns on the same day). High between day variance is attributed to differences in meteorological conditions that occurred from one day to the next. For this reason the treatment huts were evaluated day by day against their matched control. These critical findings emphasize the need for conducting extensive baseline studies through a range of environmental variables to establish movement patterns of the natural populations so that changes in these patterns can be taken into consideration upon introduction of chemical. These results also focused our attention on the need for studies that always include a control (untreated) hut paired with a treatment hut both temporally and spatially.

**Figure 3:**
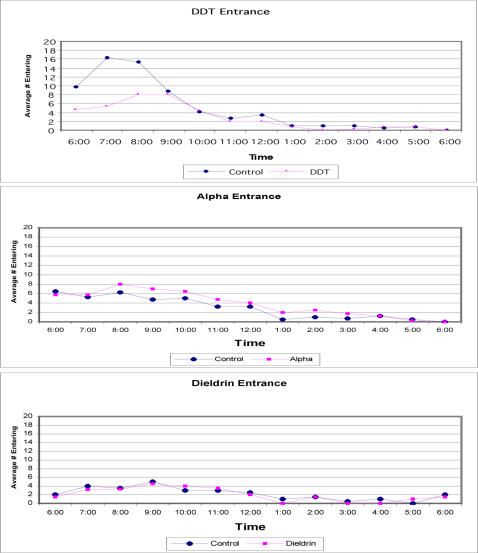
Entering *Ae. aegypti* by time for treated and matched control huts using DDT, alphacypermethrin and dieldrin.

The results showed that there were significantly fewer mosquitoes collected from the DDT treated hut compared to the control hut (*P* = 0.05). Overall, of the 400 marked *Ae. aegypti* released at the DDT treated hut, 107 (27%) were recaptured entering the hut. In comparison, 259 (65%) of the 400 marked mosquitoes released at the control hut entered the hut. This equates to a 59% reduction in numbers entering the DDT treated hut compared to the control hut. In contrast, there were no significant differences in numbers entering the alphacypermethrin treated hut compared to the paired control hut (*P* = 0.24). Actual values were 198 (50%) recaptured entering the alphacypermethrin treated hut compared to 153 (39%) entering the control hut. Dieldrin showed similar results in that there was no significant difference between the numbers of marked mosquitoes that were capture entering the treated hut as compared to the control hut. A total of 89 marked mosquitoes were collected entering the control hut as compared to 80 mosquitoes entering the treated hut. The slight reduction that was documented did not equate to a statistically significant difference and could be explained as normal background noise. The peak time of entering populations in both the control and treated huts occurred between 0800 and 0900 hrs for both the treated and the control huts (Fig 3). Differences between the treatment and control huts for time of entry were not statistically significant.

**Figure 4:**
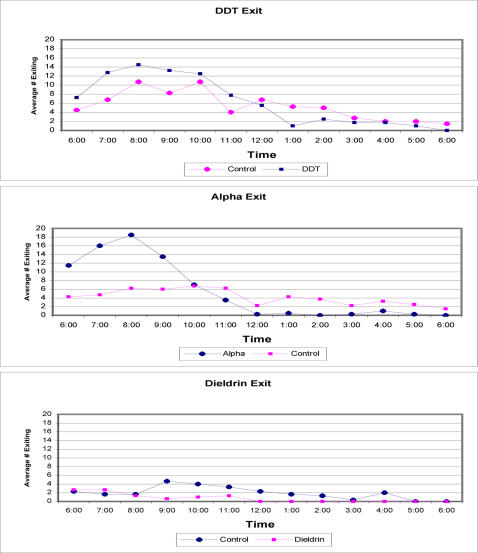
Exiting *Ae. aegypti* by time for treated and matched control huts using DDT, alphacypermethrin and dieldrin.

The time trend for the exiting populations from the DDT, alphacypermethrin and dieldrin huts with their matched controls can be seen in Figure 4. There was a 14% increase in exiting from the DDT treated hut (326 mosquitoes) compared to the control hut (281 mosquitoes). A total of 18 marked mosquitoes were collected from the floor (knockdown) in the DDT treated hut. All of these knocked down specimens were dead after a 24-h holding period. The total return in numbers exiting from the alphacypermethrin hut was 289 (72%) compared with 216 (54%) exiting from the control hut. The time trend is presented in Figure 3. Differences in numbers exiting the alphacypermethrin treated hut compared to the matched control hut equates to a 25% increase in exiting. A total of 64 marked mosquitoes were collected from the floor in the alphacypermethrin treated hut. All knocked down specimens were dead at the end of the 24-h holding period. For dieldrin, considerably more mosquitoes were collected exiting the control hut (76 females) than the treated hut (29 females). Few mosquitoes exited the dieldrin treated hut due to the toxicity of the compound in absence of any behavioral responses. A total of 138 marked mosquitoes were collected as knockdown from the floor of the dieldrin hut, all of which were dead after 24 hours. The majority of specimens on the floor were collected in the first three hours post release. After the first three hours the majority of females in the dieldrin hut had succumbed or were moribund and unable to escape. Therefore, if we evaluate the behavioral modifying actions of dieldrin during the first three hours of the collection when the greatest number of *Ae. aegypti* were still able to escape, we found very little difference in exiting between treatment (20 females) and control huts (17 females).

A total of 294 mosquitoes exited the DDT treated hut by 1200 hr with 12 knocked down inside the hut. During the first 7 hours, 207 exited the control hut, with 3 knocked down. This was an increase of 31% exiting over that observed in the control hut during the first half of the day. The results for alphacypermethrin were more dramatic. A total of 281 mosquitoes exited the alphacypermethrin hut by 1200 hr with an additional 51 mosquitoes collected in the hut as knockdown. A total of 146 marked mosquitoes were collected from the exit traps in the control huts in the first half of the collection with only 2 specimens on the floor (knocked down). This equated to a 55% increase in exiting mosquitoes by midday from the alphacypermethrin treated hut compared to the matched control hut. This same treatment of the data was not performed for dieldrin due to its extreme toxicity and lack of a behavior modifying action.

After holding mosquitoes collected from the DDT hut exit traps for 24 hrs, a total of 251 remained alive (76% survival rate) compared to 278 (99% survival rate) remaining alive for the control. Of those that were on the floor (knocked down) inside the hut, only 1 (6%) remained alive. In the alphacypermethrin hut, 205 (71% survival rate) mosquitoes removed from the traps remained alive, compared to 212 (98% survival rate) that remained alive from the control traps. All the mosquitoes that were collected in the hut that were knocked down did not recover after 24 hrs (0% survival rate).

## Discussion

This study demonstrated that the impact of insecticides on vector populations is much more complex than just toxicity. In fact, our studies showed that, while the toxic effect of a chemical like dieldrin can have a dramatic effect on the immediate population density, it carries with it the chance for rapid build up of resistance. For this reason we feel that while toxicity has an immediate impact, the long term implications make it the least important of the chemical actions. This study clearly showed that the primary indicators of chemical actions in huts were proportions repelled (spatial repellency), proportions stimulated to prematurely exit (contact irritancy), and proportions that died (toxicity). Estimates of proportions repelled were taken from hut entry data. For DDT, alphacypermethrin and dieldrin the proportions repelled were 59%, 0% and 0% respectively. Contact irritant actions were measured by numbers escaping into exit traps during the first 7 hours of observations. The proportions exiting were 31% (DDT), 55% (alphacypermethrin) and 0% (dieldrin). Toxicity was estimated from two parameters; number dead on the floor and number in exit traps that died after 24 hours. The two parameters for DDT were 5% dead on the floor, and 11% of those that escaped subsequently died. The same values for alphacypermethrin were 15% and 19%, respectively. These two parameters for dieldrin were much more dramatic at 75% dead on the floor, and 69% of those that escaped subsequently died. Numbers in exit traps that subsequently die must be separated from numbers that were irritated and exited prematurely. With no correction the mosquitoes in exit traps that died would be included in two different parameters. We address this by adding the number in exit traps that subsequently died from intoxication to the number dying from toxic actions inside the hut. Thus, toxicity included numbers that died after escaping the hut and numbers dead on the floor. To avoid inflating estimates by including mosquitoes in more than one measure, our estimate for contact irritant action included the number caught in exit traps minus the number that subsequently died.

We defined composite impact of the two chemicals by assuming that a hundred mosquitoes would enter a house, bite while indoors, and escape and survive if the house were not sprayed. We can use our proportions, described above, to evaluate how spraying with one or the other chemical will impact the 100 mosquitoes.

In huts sprayed with DDT, 59 of the 100 mosquitoes would not enter. Of the 41 that enter, 2 would die and fall to the floor. Of the 39 survivors, 12 would exit prematurely. One of the 12 mosquitoes that escaped would die within the next 24 hours. This leaves 27 mosquitoes that theoretically could bite and survive. However, it is important to understand that chemical is present in houses 24 hours each day, these statistics cover only 7 hours, not 24. These statistics suggest that DDT reduced risk from 100 mosquitoes by 73% within the first 7 hours.

In huts sprayed with alphacypermethrin, all 100 mosquitoes would enter the house. Of the 100 that entered, 15 would die. Of the remaining 85, 46 would exit prematurely and 9 of those would die. This leaves 39 mosquitoes that theoretically could bite and survive. The spatial repellent, contact irritant, and toxic actions of alphacypermethrin sum to 61% protection.

In huts sprayed with dieldrin, all 100 mosquitoes would enter the house. Of the 100 that enter, 75 would die before exiting. Of the 25 that exit, 17 would escape and subsequently die. This would leave 8 mosquitoes that could take a blood meal and survive for a summed 92% protection. While this may have an immediate impact on the population densities, it carries with it the potential for a quick build up of insecticide resistance thus rendering the chemical ineffective over time. While the failure to feed that occurs through repellency may also provide enough selection pressure to engender resistance, this phenomenon has never been documented and must be examined further.

The data presented here are based on laboratory and field tests with populations of *Ae. aegypti*. However, our earlier research showed similar but even stronger chemical actions on malaria vector mosquitoes [13], [14], [15]. Our research results were consistent with historical studies by other investigators showing that DDT exerted powerful actions on mosquito behavior [13], [15]–[17]. Those historical studies were partially reviewed in a probability model of how DDT functions in malaria control programs [18].

There has been no failure in understanding that DDT is by far the most cost-effective chemical yet discovered for sustained use in malaria control programs. However, there has been an enormous failure to accurately account for how DDT actually functions in control of malaria. As stated before, this failure has impeded the search for DDT alternatives. In a more general way, this encompasses both a failure to properly characterize and quantify the separate actions of chemicals and a failure to characterize and quantify the separate responses of vector mosquitoes to those chemical actions.

The new classification scheme that we are proposing will characterize chemicals on the basis of spatial repellent, contact irritant and toxic actions. The first criterion for evaluating a chemical is the concentration at which the chemical exceeds a threshold for vector response. If mosquitoes are intoxicated at concentrations lower than that required for a behavioral response then toxicity supersedes other actions since the insect might be overcome before being stimulated through mechanisms of contact irritancy or spatial repellency. Likewise, if an irritant response occurs at a lower concentration of chemical than required for toxicity, then the irritant response precludes toxicity since the insect or some proportion of insects may move away from the chemical before acquiring a lethal dose. These relationships are even more pronounced for a spatial repellent action. If a spatial repellent response is stimulated by a lower or equal concentration of chemical than required for either contact irritancy or toxicity, then the insect or some proportion of insects will be repelled without making contact with the chemical. Thus the three chemical actions (spatial repellent, contact irritant, toxicant) can be quantified according to proportional dose-response relationships and the relative rank order of actions can be defined.

As described, the first criterion for rank ordering of chemical actions is the relative concentration of chemical required for a given response. If a significant level of toxicity, as found with dieldrin, is produced at lower concentrations than required for contact irritant or spatial repellent actions, then toxicity is the first order action. According to this definition, a first order action occurs at lowest concentration, second order action occurs at second lowest concentration, and third order occurs at third lowest concentration. That is to say, concentration for 1st order is<concentration for 2nd order, and concentration for 2nd order is<concentration for 3rd order action.

The second criterion for evaluating a chemical is the time of contact or exposure time. Even if concentrations were equal for stimulating toxic, contact irritant, and spatial repellent responses, order could still be defined by exposure time for eliciting a given response. For example, if a contact irritant response occurred more quickly than a toxic response, the contact irritant mechanism could function to preclude toxicity by causing insects to move away from the chemical prior to acquiring a lethal dose yet may not be intense enough to prevent feeding.

The third criterion for evaluating a chemical is the combined effect of the first and second criterion (i.e. the percentage that do not enter combined with the percentage that leave prematurely). The most comprehensive ordering of chemicals would be to use both sets of parameters, chemical concentration and exposure time for a given response.

Conventional wisdom in the control of malaria vector mosquitoes is that a repellent action will neutralize the toxic effect of a compound and thus reduce the effectiveness of the chemical. This assessment is true only if we accept the notion that chemicals function to prevent malaria transmission solely by killing mosquitoes. We assert with the present study that disease transmission is prevented through breaking the man-vector contact where it occurs, inside the home. This can be done, as has occurred through the successful use of DDT, by creating a spatial repellent barrier that precludes a large proportion of the mosquitoes from entering the house as well as serves as a contact irritant for those that do enter, causing them to potentially leave without taking a blood meal.

Considerable effort has been expended to define levels of toxicity based on standard exposure times. The focus has been to work with varying concentrations for a fixed exposure to define concentrations for mortality. Little effort has been devoted to the question of exposure time as a variable for a standard concentration. From the very beginning of DDT use, there was recognition that mosquitoes must be in physical contact with the chemical for 20 minutes or more for acceptable levels of mortality (50% and higher) [19]. This requirement was recognized as a problem because both field and laboratory data showed that mosquitoes were quickly repelled by the chemical. Our laboratory behavioral assays were conducted for only 10 minutes, and yet we still were able to quantify a spatial repellent response. We have no detailed exposure time analyses for the set of data presented here. However, our past studies showed behavioral responses to occur immediately and Kennedy's classic study published in 1947 suggested that some behavioral responses occurred almost instantaneously with chemical exposure. This subject warrants a great deal more research, and exposure time could be an important component of any comprehensive rank ordering of chemical actions.

The field studies showed that the new assay system precisely discriminates between spatial repellent, contact irritant and contact toxic actions of test compounds. The findings from both laboratory and field studies showed that spatial repellency is the first order action of DDT. Furthermore, tests showed that contact irritancy is the second order action and that toxicity is only a third order action. As a killing agent, DDT is inferior to modern insecticides which kill mosquitoes more quickly and at much lower concentrations. Even Mueller in his original discovery recognized that DDT was a very slow-acting insecticide [20]. This rank ordering of DDT actions is entirely consistent with decades of published laboratory and field studies on DDT actions. Alternatively, our data showed alphacypermethrin to function primarily as a contact irritant and secondarily as a toxicant, but it does not elicit a significant spatial repellent response with *Ae. aegypti* mosquitoes. Prior hut studies against malaria vectors confirmed these findings with alphacypermethrin as general characteristics of pyrethroid insecticides [15]. Dieldrin functioned primarily as a toxicant but shows no behavior modifying actions, which seems to increase its killing potential.

As stated above, the historical record of malaria control operations show that DDT is the most cost-effective chemical for malaria control. Even now DDT is still considered to be the cheapest and most effective chemical for use in house spray operations. Its long residual action when sprayed on inner walls further enhances its cost-effectiveness. These facts illustrate a remarkable paradox. The paradox is that DDT is widely considered to be the most effective chemical for malaria control and, unfortunately, is widely considered to have no important function other than killing mosquitoes. Yet, DDT does not provide quick knockdown or high mortality to mosquitoes. Observations reported here provide a new and clearer explanation of how DDT actually functions to control malaria transmission inside houses. The data obtained from both laboratory and field studies on the chemical actions of DDT confirm the probability model of Roberts *et al.* [18] which explains the roles of repellency and contact irritancy in disrupting malaria transmission. Success through the mechanism of spatial repellency means that DDT basically functions as a form of chemical screening, which stops mosquitoes from entering houses and transmitting malaria.

Alphacypermethrin, on the other hand, is primarily a contact irritant and a toxicant as exhibited by the pronounced exiting response and high knockdown in laboratory assays and inside the huts. However, this compound did not elicit a repellent response from the mosquitoes under controlled laboratory conditions or repel mosquitoes from entering the hut in the field. The mosquitoes could still enter and bite unprotected inhabitants, thereby transmitting disease. Furthermore, most mosquitoes that did enter were able to leave the hut without picking up a lethal dose of the compound. While this is documented as a premature exiting behavior, the mosquitoes were still present in the hut for a short time and could have potentially acquired a blood meal if one had been accessible.

Dieldrin is primarily a toxicant but does not irritate or repel *Ae. aegypti*. It is important for use to take a closer look at how this combination of actions effect the impact dieldrin has on vector populations. This compound fits all of the characteristics of an ideal insecticide, i.e. it is a strong toxicant that does not modify insect behavior. Our data indicate that the mosquitoes will sit on the treated surface without becoming irritated, thereby picking up a lethal dose resulting in a rapid reduction in the adult female populations. While this may have an immediate impact on the population densities, it carries with it the potential for a quick build up of insecticide resistance. If we look to the history of dieldrin use, we find that this is precisely what happened when this chemical was applied. Ascher in 1955 [21] cited examples of the extremely rapid development of dieldrin-resistance in insects not previously resistant. Brown in 1958 [22] observed the rapid development of resistance in *An. gambiae* as soon as dieldrin was used. While the immediate toxic action is beneficial, the long term impact is the rapid build up of resistance thus rendering the chemical ineffective.

Existing criteria for dealing with insecticide resistance have resulted in countries abandoning DDT when vectors became resistant to the insecticide's toxic actions. The criteria include no allowance for the possibility that mosquitoes might become resistant to toxic actions and still be susceptible to a chemical's spatial repellent or contact irritant actions. Given that spatial repellent action is the first order action of DDT residues, resistance to a toxic action may not signify that DDT will no longer exert control over malaria transmission. The populations of *Ae. aegypti* used in our studies were DDT resistant. Yet, the spatial repellent responses that we documented were very similar to those Kennedy reported for *Ae. aegypti* in 1947 [1]. Resistance to a toxic action seems to have no influence on the behavioral responses of mosquitoes to spatial repellent or contact irritant actions.

To date, a truly efficacious DDT replacement has not been found and one may never be found because of the true nature in which DDT functions. Success through the mechanism of spatial repellency means that DDT functions as a form of chemical screening, which stops mosquitoes from entering houses and thus breaks the man/vector contact at its most critical point: when people are sleeping in their homes. DDT's secondary action stimulates those mosquitoes that do enter to prematurely exit, potentially without biting and transmitting disease. Toxicity is only a third order action of DDT and it is considered to be a very poor killing agent. We propose that a search for a DDT replacement should focus on a new set of selection criteria of spatial repellency, contact irritancy and toxicity. We must move from selecting vector control chemicals solely on the basis of toxicity and accept a new paradigm of selection criteria focused on multiple chemical actions for the control of disease transmission by breaking man-vector contact.
